# Relative validity and reliability of a quantitative food frequency questionnaire for adults in Guam

**DOI:** 10.3402/fnr.v59.26276

**Published:** 2015-05-05

**Authors:** Rachael T. Leon Guerrero, Marie Chong, Rachel Novotny, Lynne R. Wilkens, Grazyna Badowski, Michelle Blas-Laguana, Suzanne Murphy

**Affiliations:** 1 College of Natural & Applied Sciences, University of Guam, Mangilao, Guam; 2 College of Tropical Agriculture and Human Resources, University of Hawaii at Manoa, Honolulu, Hawaii; 3 University of Hawaii Cancer Center, Honolulu, Hawaii

**Keywords:** food frequency questionnaire, dietary assessment, relative validation, Guam

## Abstract

**Background:**

Guam is a US territory in the western Pacific with a diverse population that includes understudied ethnic groups such as Chamorros and Filipinos. A food frequency questionnaire (FFQ) to estimate dietary intake was needed to facilitate studies of diet and health among adults living in Guam.

**Objective:**

To develop and validate an FFQ to assess dietary intake over a 1-year period among adult Guam residents.

**Design:**

A three-part study was conducted: 1) an initial cross-sectional study using 24-h recalls to identify a food and beverage list for the FFQ and resulting in a final FFQ containing 142 food and drink items; 2) to test reliability, 56 different individuals completed the FFQ twice; and 3) to test relative validity, self-administered FFQs and up to 2 days of food record data from an additional 109 individuals were collected, and daily nutrient intake from the two methods was compared.

**Results:**

The reliability of the FFQ was very good (*ρ* range=0.65–0.75), and the relative validity of the FFQ was good for women (median Spearman's correlation [*ρ*] between instruments of 0.45 across 20 nutrients and an interquartile range [IQR] of 0.42–0.58) and generally adequate for men (median *ρ=*0.31, IQR=0.23–0.55). Validity was also good for Chamorros (median *ρ=*0.47, IQR=0.38–0.53) and generally adequate for Filipinos (median *ρ=*0.42, IQR=0.20–0.62). Correlations after energy adjustment were lower (overall median *ρ=*0.20, IQR=0.14–0.26).

**Conclusions:**

The FFQ can be used to rank nutrient intake for adults in Guam and may be helpful in the analysis of relationships between diet and chronic disease in Guam.

Guam, a US territory, is located in the tropical Mariana Islands of the western Pacific. Currently, there are no US national food intake data collected in Guam, such as the National Health and Nutrition Examination Survey (1). Further, the ethnic composition of the population is distinct, with native Chamorro populations and a sizeable Filipino population (2, 3). In addition, the food environment is diverse with a large quantity of food imports and various indigenous tropical foods. Thus, an FFQ created for the United States may not adequately capture dietary intake in Guam. An FFQ is important for use in research studies to examine associations between diet and chronic disease. Approximately 60% of deaths on Guam are caused by chronic diseases linked to poor diet and lifestyle patterns (4); therefore, an FFQ designed to assess dietary intake of residents living in Guam would be useful for studying these long latency conditions.

The diet of early Chamorros, the natives of Guam and the Northern Mariana Islands, was predominantly plant-based and included taro, yams, breadfruit, bananas, cassava, coconut, and fish (5). After World War II, the Chamorro diet began to shift from locally grown foods to imported rice and highly processed canned goods, such as Spam^®^, corned beef, and vienna sausage. Early dietary assessments of adults on Guam, conducted more than 25 years ago (6–8), documented this continued shift from locally grown foods to highly processed imported foods. A recent study, using 24-h recalls to assess dietary intake, showed that Chamorro adults consumed significantly more sugar-sweetened beverages and had diets with significantly higher energy density than Filipino adults on Guam (9).

The current population of Guam is characterized by unique ethnic variation (2): 42% Chamorro, 33% Filipino, 7% white, 6% Asian, and 8% other Pacific Islanders. An updated dietary assessment tool is needed to assess longer-term dietary intake among people living in the Mariana Islands. To fill this gap, we developed an FFQ to estimate dietary intake over the past year in this population.

There are a variety of methods available to assess dietary intake, and the FFQs are the most convenient and widely used in epidemiological studies because they do not need to be administered by trained personnel, are fairly inexpensive, and can be self-administered (10, 11). Because FFQs are designed to be culturally and ethnically sensitive, the validation and reproducibility of an FFQ needs to be determined each time a tailored FFQ is used for a new target population (12), and foods consumed by that population must be incorporated. A drawback to using an FFQ is that, compared to diet records and recalls, there needs to be sufficient previous research to generate a representative food list, and a participant is not able to be as specific about food consumption, food preparation, and food amounts (12). Another drawback is that the FFQ solicits information on dietary intake over a less precisely defined period of time (12). FFQs have been found to reasonably rank individuals but not to provide very accurate measures of absolute diets. That is, nutrients from FFQs are generally subject to random and systematic error. On the other hand, processing data from a FFQ can be faster and, therefore, more cost-effective for a population-based study (12). There is no ‘gold standard’ to validate all aspects of a quantitative FFQ, and its relative validity is generally determined by comparing the results of the FFQ instrument to the results of other dietary assessment methods or to biomarkers (13). Because it was beyond the scope of the present study to use a biomarker as a comparison for the FFQ, we used 1-day and 2-day food records as the dietary assessment method for comparison in this study. The methodology was based on that used for the Multiethnic Cohort (MEC) Study in Hawaii (14, 15). Therefore, the aim of the present study is to assess the relative validity of the 142-item FFQ against food records and to determine its reliability. Ways to improve the FFQ will be identified based on the results of these assessments.

## Materials and methods

### Development of the FFQ

In 2004, the University of Guam Cancer Research Center and the University of Hawaii Cancer Center (UHCC) conducted a cross-sectional study of 127 adults living in Guam, aged 25–65 years, who were either of Chamorro (*n=*66) or Filipino (*n=*61) ethnicity. Sampling procedures and results have been described elsewhere (9). Briefly, a 24-h dietary recall was conducted using the modified 3-pass method (16), and nutrient analysis was performed using the Pacific Tracker (PacTrac) program, with the 4th edition food composition table (FCT) (17, 18).

**Fig. 1:**
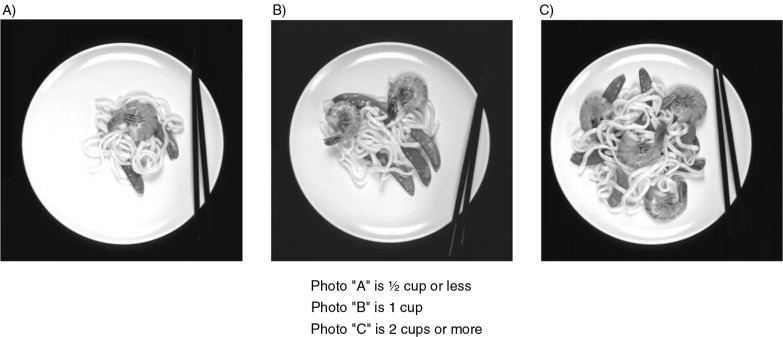
Sample portion size photos of foods on the food frequency questionnaire.

A complete list of all the foods and beverages recorded from the 24-h recalls served as the preliminary food list for the FFQ. Additional foods that had not appeared in the recalls but were considered relevant, such as locally grown produce (e.g. breadfruit, taro leaves, and green papaya) and common ‘fiesta’ or ‘celebration foods’ (e.g. roast pig; red rice, which is unenriched white rice cooked in annatto-colored water; and kelaguen, which is finely shredded chicken mixed in a spicy sauce made with lemon) (19), were added to the draft FFQ. The FFQ format utilized was very similar to the format of the successful MEC FFQ (14, 15). For each food item, three commonly used portion sizes, similar to those used in the MEC FFQ, were listed along with a question asking how often, on average, the food was consumed during the past year. Each food item had eight frequency responses, which ranged from never (less than once per month) to two or more per day. Questions regarding dietary supplement use and cooking methods were included. This preliminary FFQ was reviewed and pretested by 10 individuals (self-administered) to check for grammatical errors, as well as for missing foods. Photographs of representative food items, showing three different portion sizes, were then added to the FFQ to facilitate quantification of intakes (Fig. 1).

### Data collection

The Committee on Human Research Subjects at the University of Guam approved both the reliability and validity studies.

#### FFQ reliability test

In order to test the reliability of the FFQ, 56 individuals from Guam volunteered to complete the FFQ via self-administration on two separate occasions, 4 weeks apart. These participants were not part of previous studies. Participants were self-identified as Chamorro (*n=*33), Filipino (*n=*20), or white (*n=*3) and were between the ages of 18–35 years. After participants completed both FFQs, a research assistant double-checked the FFQ for missing values and then coded each FFQ. All coded FFQs were hand-entered into an Excel spreadsheet. To ensure accuracy of entry, all FFQs were entered twice and then entries were crosschecked and corrected.

#### FFQ relative validation

In order to test the relative validity of the FFQ, 109 individuals from Guam were asked to complete the FFQ via self-administration and also to complete a 2-day food record. These participants were not part of the previous studies or the FFQ reliability test and were recruited via newspaper advertisements, flyers, social media, and word of mouth. Male (*n=*43) and female (*n=*66) participants were predominantly Chamorro (*n=*70) or Filipino (*n=*35) and were 18–61 years of age.

A trained research assistant met with participants who agreed to complete a food record and the FFQ. The participants were asked to complete the food records for 2 days, but a single day of recording was accepted. The research assistant reviewed the methods for completing the food record and showed the participant a sample of a completed record. Each participant was asked to record all foods and beverages consumed and to either measure or estimate the portion sizes based on standard household measures. Participants were also instructed how to complete the FFQ after the food record. Participants reported that it took approximately 30–40 min to complete the FFQ. Once the food record and FFQ were completed and returned, the registered dietitian and a research assistant double-checked information about missing data. Of the 109 participants, all completed the FFQ and 63 completed the 2-day food record; the remaining 46 participants completed only a 1-day food record.

### Analyses

Data analyses were performed using SAS version 9.2 (SAS Institute, Cary, NC, USA) and Statview (Abacus Concepts, Inc. 1995, version 4.5, Berkeley, CA) statistical packages. A *p* value of less than 0.05 was considered statistically significant.

#### Computation of daily nutrient intake from the food record

All food record data were coded, entered, and analyzed using the PacTrac Program, 4th edition (16, 17). PacTrac was developed at UHCC to analyze typical diets in Hawaii. Version 4 of the FCT in PacTrac was used, which added 85 recipes from the Mariana Islands to ensure that the database included foods commonly consumed by Chamorro and Filipino residents on Guam and Saipan. Dietary components were computed separately by day for records containing 2 days of data.

#### Computation of daily nutrient intake from the FFQ using an FCT

An FCT was constructed specifically for the FFQ. Most of the 142 items on the FFQ were aggregated food and beverage items rather than individual foods and beverages. The base food composition data provides the amounts of each nutrient per 100 g of food for individual foods and beverages rather than for recipes or mixed drinks. Therefore, FCT records were created for the 142 FFQ items as weighted averages of the FCT records for the individual foods assigned to that item, where the weights were the number of times the foods were consumed in the original 127 24-h recalls. A summary file was created with one record for each of the 142 FFQ items that had the weighted averages of the nutrient values for the foods assigned to the item. The base food composition data for the FFQ FCT and PacTrac were primarily based on the US Department of Agriculture's National Nutrient Database for Standard Reference, Release 18, supplemented with data from other research and commercial publications, in addition to the information specific to Hawaii and Guam.

#### Nutrient computation for each individual

Daily nutrient intake from each of the 142 FFQ food items was determined for each subject in the reliability and validity studies. Participants selected the frequency of consumption from one of eight categories: never or hardly ever, once a month, two to three times a month, once a week, two to three times a week, four to six times a week, once a day, and two or more times a day. The frequency categories were converted to daily frequencies by dividing monthly frequencies by 30.4 days and weekly frequencies by 7 days. Gram weights for the three portion sizes for each FFQ item were estimated using the procedures developed for the MEC FFQ (14, 15), which were based primarily on the weight of the pictured items and the descriptions given, for example, ‘one chicken drumstick’. For each person, the daily gram intake of each of the 142 food items was calculated as the product of the times per day consumed and the grams per selected portion size. The daily gram intakes were then multiplied by the amount of nutrient per gram of the food item. The total daily nutrient intake for each participant, as measured by the FFQ, was obtained by summing the intake of each nutrient across the 142 food items.

#### Dietary components

For each day of records in the FFQ, the following dietary components were included in the analysis: energy, protein, total fat and fat components, carbohydrate, dietary fiber, calcium, phosphorus, magnesium, iron, zinc, and sodium. For each component, other than energy, an energy-adjusted density value was computed. For macronutrients (protein, carbohydrate, fat, and saturated fat), we computed the percent of energy contributed by that macronutrient. For all other components, a nutrient density was computed as the daily amount of the component divided by the daily energy, multiplied by 1,000. The results using energy adjustment by the residual method (12) were similar and not reported.

#### Statistical methods

*FFQ reliability*. In order to measure concordance between the first and second FFQs administered to the same participant at different times, Spearman's rank correlation coefficients (*ρ*) were computed comparing energy and 19 nutrients between FFQ administrations.

*FFQ relative validation*. Mean nutrient intakes were presented and compared by instrument, overall, and by sex and by race/ethnicity. Relative concordance, such as the ability to rank individuals correctly, was assessed between sex/race/age adjusted nutrient intakes from the 142-item FFQ and the food records. The adjusted values were computed as the residuals of a regression of nutrient on a sex indicator, a race indicator (Chamorro, other), and age. For individuals with 2 days of food records, an average across days was used as the intake value. If both days were weekdays or weekend days, a simple average was used. If both a weekday and a weekend day were present, the average was weighted by 5/7 for weekdays and 2/7 for weekend days to better reflect the weekly intake. Spearman's correlations (*ρ*) were computed and corrected for within-person day-to-day variability by multiplying by the square root of one plus the attenuation factor *λ* for that nutrient (12). The attenuation factor was computed as $\lambda =(\sigma_{W}^{2}/\sigma_{B}^{2})$, where $\sigma_{W}^{2}$ and $\sigma_{B}^{2}$ are the within- and between-person variances from a mixed model of the log-transformed nutrient for the 2 days of records among the 63 individuals. Spearman's correlations were also computed between nutrients within instruments.

## Results

### Reliability

Table 1 presents the Spearman's correlation coefficients (*ρ*) estimating reliability. For energy and macronutrients, crude correlation coefficients ranged from 0.65 (carbohydrate) to 0.71 (energy, protein, and fat). For micronutrients, crude correlation coefficients ranged from 0.66 (vitamin B12) to 0.75 (vitamin C). Energy-adjusted correlation coefficients were lower, ranging from 0.32 (carbohydrate) to 0.71 (dietary fiber).

**Table 1:** Correlations between the first and second daily nutrient intakes from food frequency questionnaires among participants (*n =* 56) in the reliability study

Daily nutrient intake	Spearman's *ρ*	Spearman's *ρ* for energy-adjusted nutrient intake^a^
Energy (kcal)	0.71	
Protein (g)	0.71	0.33
Total fat (g)	0.71	0.35
Carbohydrate (g)	0.71	0.32
Dietary fiber (g)	0.73	0.71
Calcium (mg)	0.71	0.70
Phosphorus (mg)	0.69	0.62
Magnesium (mg)	0.70	0.71
Iron (mg)	0.68	0.54
Zinc (mg)	0.67	0.32
Sodium (mg)	0.71	0.41
Potassium (mg)	0.72	0.64
Thiamin (mg)	0.70	0.54
Riboflavin (mg)	0.72	0.60
Niacin (mg)	0.68	0.49
Vitamin B6 (mg)	0.70	0.54
Vitamin B12 (µg)	0.66	0.43
Vitamin C (mg)	0.75	0.69
Saturated fat (g)	0.71	0.49
Monounsaturated fat (g)	0.70	0.36
Polyunsaturated fat (g)	0.69	0.41

a Partial correlations adjusted for sex and race/ethnicity.

### Relative validity

Table 2 presents the mean and standard deviation of daily nutrient intake from the food records and the FFQ, as well as the Spearman's correlation coefficients (*ρ*) between these two methods. The mean daily intake of several nutrients (total fat, niacin, and monounsaturated fat) from the FFQ was not significantly different from intake as measured by the food records; however, the mean intake of the remaining nutrients from the FFQ was significantly higher than those of the food records (*p*≤0.05). FFQs are generally used to rank the intakes of individuals rather than to give absolute amounts. According to Segovia-Siapco et al. (20), it is common for FFQs with more than 100 food items to overestimate dietary intake compared to other dietary assessment methods. For energy and macronutrients, the Spearman's rank correlations varied between 0.22 for polyunsaturated fat to 0.61 for dietary fiber. For micronutrients, the Spearman's rank correlations varied between 0.28 for niacin to 0.67 for riboflavin. The median correlation between the instruments was 0.38, and the IQR was 0.30–0.54. The correlations after energy adjustment were lower, with a median of 0.20 and an IQR of 0.14–0.26.

**Table 2:** Comparison of mean daily nutrient intake and concordance between the food records and food frequency questionnaire (*n=*109)

Daily nutrient intake	Food frequency questionnaire Mean±SD	Food records Mean±SD	*P*	Spearman's rank correlation for nutrient intake*	Spearman's rank correlation for energy-adjusted nutrient intake*
Energy (kcal)	2860±1746	2118±842	<0.0001	0.36	–
Protein (g)	112±71	96.5±41.2	0.031	0.38	0.44
Total fat (g)	98.9±67.7	86.3±43.3	0.106	0.29	0.10
Carbohydrate (g)	383±234	239.0±109.2	<0.0001	0.37	0.21
Dietary fiber (g)	26.0±18.0	14.8±11.2	<0.0001	0.61	0.43
Calcium (mg)	1077±895	643.8±437.7	<0.0001	0.58	0.14
Phosphorus (mg)	1696±1106	1229.3±516.2	<0.0001	0.67	0.46
Magnesium (mg)	432±273	273.1±127.4	<0.0001	0.44	0.16
Iron (mg)	24.3±14.9	16.9±8.8	<0.0001	0.55	−0.07
Zinc (mg)	15.5±9.7	13.2±5.9	0.017	0.49	−0.02
Sodium (mg)	3969±2579	3373.7±1872.9	0.022	0.30	0.49
Potassium (mg)	3777±2542	2327.2±1114.6	<0.0001	0.54	0.12
Thiamin (mg)	2.2±1.3	1.7±0.7	<0.0001	0.47	0.16
Riboflavin (mg)	2.7±1.9	1.9±0.9	<0.0001	0.67	0.27
Niacin (mg)	33.9±20.9	27.3±12.0	0.003	0.28	0.19
Vitamin B6 (mg)	3.0±1.9	2.1±1.2	<0.0001	0.34	0.20
Vitamin B12 (µg)	10.4±9.2	6.0±10.7	0.001	0.38	0.21
Vitamin C (mg)	187±145	101.2±111.1	<0.0001	0.51	0.23
Saturated fat (g)	33±24	26.8±15.6	0.009	0.28	0.04
Monounsaturated fat (g)	35.7±24.6	34.0±17.3	0.530	0.26	0.25
Polyunsaturated fat (g)	21.4±14.8	18.1±11.4	0.046	0.22	0.17
Median				0.38	0.20
Interquartile range				0.30–0.54	0.14–0.26

* The correlations were adjusted for day-to-day variability of the food records by dividing by an attenuation factor computed as the square root of the ratio of within- to between-person variances based on data from 2 days of food records. An average across days was used as the food record nutrient when 2 days of data were available.

Table 3 shows the results of Spearman's correlation coefficients (*ρ*) computed between the food records and FFQ by sex and race/ethnicity. For females, there was generally good concordance between the food records and FFQ, with correlation values for macronutrients ranging between 0.35 for saturated fat and 0.69 for dietary fiber and correlation values for micronutrients ranging between 0.30 for vitamin B12 to 0.75 for iron. The median correlation value for females was 0.45, with an IQR of 0.42–0.58 for (absolute) nutrient intakes, and 0.19 (IQR: 0.12–0.35) for nutrient densities. For males, there was lower concordance between the food records and FFQ, with correlation values for macronutrients ranging between –0.09 for polyunsaturated fat and 0.46 for dietary fiber and correlation values for micronutrients ranging between 0.14 for niacin to 0.66 for vitamin B12. The median correlation value for males was 0.31 (IQR: 0.23–0.55) for nutrient intakes and 0.20 (IQR: 0.14–0.26) for nutrient densities. For Chamorros, there was generally good concordance between the food records and FFQ for most nutrients, with correlation values for macronutrients ranging between 0.35 for protein and 0.71 for dietary fiber and correlation values for micronutrients ranging between 0.21 for vitamin B12 to 0.72 for riboflavin. The median correlation value for Chamorros was 0.47 (IQR: 0.38–0.53) for nutrient intakes and 0.29 (IQR: 0.17–0.46) for nutrient densities. For Filipinos, there was poor concordance for total fat and fat components and reasonable concordance for other macronutrients, with correlation values ranging between 0.17 for energy and 0.45 for protein and correlation values for micronutrients ranging between 0.20 for sodium to 0.72 for phosphorus. The median correlation value for Filipinos was 0.42 (IQR: 0.20–0.62) for nutrient intakes. The correlations for nutrient densities were poor, with a median of –0.01.

**Table 3:** Spearman correlation coefficients (*ρ*)* between the daily nutrient intakes from the diet records and the food frequency questionnaire by sex and race/ethnicity

Daily nutrient intake	Males (*n=*43)	Females (*n=*66)	Chamorro (*n=*70)	Filipino (*n=*35)
Energy (kcal)	0.26	0.45	0.53	0.17
Protein (g)	0.26	0.42	0.35	0.45
Total fat (g)	0.16	0.44	0.49	−0.12
Carbohydrate (g)	0.30	0.46	0.47	0.20
Dietary fiber (g)	0.48	0.69	0.71	0.42
Calcium (mg)	0.55	0.61	0.64	0.58
Phosphorus (mg)	0.62	0.67	0.59	0.72
Magnesium (mg)	0.45	0.47	0.47	0.44
Iron (mg)	0.23	0.75	0.53	0.51
Zinc (mg)	0.36	0.56	0.38	0.71
Sodium (mg)	0.18	0.38	0.38	0.20
Potassium (mg)	0.56	0.56	0.51	0.64
Thiamin (mg)	0.34	0.58	0.57	0.39
Riboflavin (mg)	0.62	0.70	0.72	0.70
Niacin (mg)	0.14	0.38	0.30	0.35
Vitamin B6 (mg)	0.31	0.37	0.38	0.27
Vitamin B12 (µg)	0.66	0.30	0.21	0.62
Vitamin C (mg)	0.61	0.42	0.50	0.65
Saturated fat (g)	0.24	0.35	0.46	−0.04
Monounsaturated fat (g)	0.07	0.43	0.44	−0.14
Polyunsaturated fat (g)	−0.09	0.42	0.46	0.01
Median	0.31	0.45	0.47	0.42
Interquartile range	0.23–0.55	0.42–0.58	0.38–0.53	0.20–0.62

* The correlations were adjusted for day-to-day variability of the food records by dividing by an attenuation factor computed as the square root of the ratio of within- to between-person variances based on data from 2 days of food records. An average across days was used as the food record nutrient when 2 days of data were available.

To further understand the suboptimal correlations for dietary fat in men and Filipinos, a sensitivity analysis was performed where subsets of individuals who strongly influenced the correlations were removed. We identified individuals who had the largest differences for dietary fat component intakes between instruments and searched for the foods that were major contributors of their dietary fat intake in the FFQ and records. The individuals with the largest differences between instruments were found to have large and implausible levels of intakes on the FFQ for foods that had several varieties listed, such as chicken not in a mixed dish (three items), mixed dishes with vegetables (four items), rice (three items), and pasta (two items). When the 8 men with extreme values for these items (compared to the records) were excluded, the correlations between instruments overall and for men and Filipinos improved; Table 4 shows the results of Spearman's correlation coefficients (*ρ*) between the food records and FFQ by sex and race/ethnicity after the 8 men were excluded from the analysis. No particular subset of women was found based on the differences that improved the concordance between methods, and therefore none were excluded. For all participants excluding the eight extremes, the Spearman's rank correlations for macronutrients varied between 0.41 for saturated fat to 0.69 for dietary fiber. For micronutrients, the Spearman's rank correlations varied between 0.43 for niacin to 0.75 for riboflavin. The median correlation between the instruments was 0.51, and the IQR was 0.44–0.61. The correlations after energy adjustment were still considerably lower, with a median of 0.20 and an IQR of 0.05–0.34. For males, the concordance was good, with correlation values for macronutrients ranging between 0.51 for saturated fat and 0.76 for dietary fiber; correlation values for micronutrients ranged between 0.41 for sodium to 1.00 for phosphorus. The median correlation value for males was 0.65 (IQR: 0.62–0.76) for (absolute) nutrient intakes and 0.30 (IQR: −0.01–0.50) for nutrient densities. For Filipinos, the concordance also improved, with correlation values for macronutrients ranging between 0.11 for monounsaturated fat and 0.57 for dietary fiber; correlation values for micronutrients ranged between 0.23 for vitamin B6 to 0.84 for riboflavin. The median correlation value for Filipinos was 0.59 (IQR: 0.40–0.65) for nutrient intakes. The correlatons for nutrient densities remained poor.

**Table 4:** Spearman correlation coefficients (*ρ*)* between the diet records and the food frequency questionnaire in a sensitivity analysis with outliers removed^a^

Daily nutrient intake	Overall (*n=*101)^a^	Males (*n=*35)^a^	Females (*n=*66)	Chamorro (*n=*70)	Filipinos (*n=*31)^a^
Energy (kcal)	0.50	0.63	0.45	0.53	0.42
Protein (g)	0.54	0.70	0.42	0.35	0.59
Total fat (g)	0.49	0.62	0.44	0.49	0.24
Carbohydrate (g)	0.43	0.42	0.46	0.47	0.40
Dietary fiber (g)	0.69	0.76	0.69	0.71	0.57
Calcium (mg)	0.59	0.56	0.61	0.64	0.63
Phosphorus (mg)	0.79	1.00	0.67	0.59	0.88
Magnesium (mg)	0.58	0.81	0.47	0.47	0.52
Iron (mg)	0.70	0.62	0.75	0.53	0.70
Zinc (mg)	0.68	0.98	0.56	0.38	0.74
Sodium (mg)	0.43	0.41	0.38	0.38	0.65
Potassium (mg)	0.61	0.69	0.56	0.51	0.68
Thiamin (mg)	0.61	0.71	0.58	0.57	0.62
Riboflavin (mg)	0.75	0.82	0.70	0.72	0.84
Niacin (mg)	0.43	0.68	0.38	0.30	0.45
Vitamin B6 (mg)	0.45	0.68	0.37	0.38	0.23
Vitamin B12 (µg)	0.42	0.65	0.30	0.21	0.65
Vitamin C (mg)	0.58	0.83	0.42	0.50	0.65
Saturated fat (g)	0.41	0.51	0.35	0.46	0.25
Monounsaturated fat (g)	0.47	0.63	0.43	0.44	0.11
Polyunsaturated fat (g)	0.46	0.63	0.42	0.46	0.38
Median	0.51	0.65	0.45	0.47	0.59
Interquartile range	0.44–0.61	0.62–0.76	0.42–0.58	0.38–0.53	0.40–0.65

* The correlations were adjusted for day-to-day variability of the food records by dividing by an attenuation factor computed as the square root of the ratio of within- to between-person variances based on data from 2 days of food records. An average across days was used as the food record nutrient when 2 days of data were available.

a Eight male outliers were removed because they reported excessive amounts for food items with several types listed in food frequency questionnaire, such as chicken, mixed dishes with vegetables, rice, and pasta.

## Discussion

The aim of this study was to evaluate the reliability and relative validity of the 142-item FFQ for healthy adults living in Guam and also to find ways to improve the questionnaire. This FFQ was designed to quickly assess daily intake of macro- and micronutrients, based on 142 food items. Willett (12) has suggested that excessively long FFQs can negatively affect validity due to respondent fatigue and boredom. However, Cade et al.'s (13) review of FFQs published between 1980 and 1999 reported a median number of 79 food items, with a range of 5–350 food items for FFQs. The 142-item FFQ used in the present study has fewer food items than many other FFQs, such as the MEC study (14) and others that ranged from 150–276 food items (21–26); it is on the shorter side of an acceptable length. Also, the ethnically diverse population of Guam required inclusion of different food items that were important to the diet for the different cultural groups. Addition of even more cultural food items might improve a future version of the questionnaire.

In order to avoid major shifts in dietary habits, we chose a short period between the two FFQ administrations to assess the reliability of the questionnaire in this study. Overall, correlations for the reproducibility study were within an acceptable range. According to Willett (12), reproducibility studies with correlation coefficients between 0.5 and 0.7 are typical and acceptable. The correlation coefficients in the reproducibility study ranged from 0.65 for carbohydrate to 0.75 for vitamin C, with a median of 0.70.

As the reference tool, the mean of 1-day or 2-day diet records were used for evaluation of relative validity of the FFQ. No dietary assessment tool is a gold standard for measuring all aspects of dietary intake. However, diet records are considered an available and feasible reference tool for validating FFQs. Diet records have limitations; they place a burden on participants, which can lead to a poor response rate, and the process of keeping a record may alter intake substantially from usual intake (12). However, diet records are considered more appropriate, as a reference method, compared to recalls because records do not rely on memory and conceptualization of portion sizes, likely leading to fewer correlated errors with the FFQ (12). Several biomarkers are good measures of true nutrient intake, but they are costly; therefore it was beyond the scope of the present study to use biomarkers to validate the FFQ.

Correlation results for relative validity for women and Chamorros were found to be within the generally accepted ranges of 0.5–0.7 and are comparable to those of similarly conducted studies (12). For men and Filipinos, the correlations were also reasonable for all nutrients except fat components, where the concordance was poor. It was found that some individuals had difficulty correctly adjusting consumption frequencies of food that had several types listed on the FFQ, a task that was particularly difficult for young Filipino men. For example, one such individual reported eating a medium portion of each variety of chicken (fried chicken, BBQ chicken, roasted/baked/grilled/skewered chicken) 2–3 times a week, while the 24-h recalls did not display chicken consumption each day. When individuals who were believed to have overestimated these items were removed, the correlations were in the acceptable range for all groups. To improve the quality of information from the FFQ, it is recommended either that the FFQ be administered by an interviewer or that it be reviewed with the participant after completion, in order to check overall consumption of these items. Subsequent versions of the FFQ will include questions about overall consumption of chicken, mixed dishes, rice, and pasta, in addition to questions by type. These types of questions are included in the Block questionnaire for fruits and vegetables (27, 28), which allows for adjustment for overestimation. The FFQ was valid and reliable for females and Chamorros and with reasonable effort can be made so for males and Filipinos.

The correlations between instruments for energy-adjusted nutrients were substantially lower than the correlations for (absolute) nutrient intakes, for both the relative validation and reliability studies. This finding is in contrast with most other validation studies, which find that adjustment for energy improves agreement, by accounting for the completeness of the FFQs and food records (14, 15), and reduces measurement error (29, 30). A possible explanation is that the diets of people on Guam tend to be quite energy-dense and most macronutrients are consumed from a few foods. In a previous study, we found that adults on Guam had a high dietary energy density value of 1.74 kcals/g (9); this value is much higher than the public health goal for dietary energy density of 1.25 kcals/g recommended by the most recent report from the World Cancer Research Fund/American Institute for Cancer Research (31). A plausible explanation is that many of the energy-dense foods consumed in Guam provide high levels of protein, fat, and carbohydrate. As an illustration, among the top 20 food items contributing to energy in the 109 individuals, 14 (70%) were also among the top 20 food items contributing to at least two of the three macronutrients (i.e. fat, protein, and carbohydrate). Examples include fried rice, fried chicken, and macaroni and cheese. This contrasts with the MEC, where only 9 (45%) of the top 20 energy-contributing food items were among the top contributors of more than one of the three macronutrients; these included bread items and milk. In the MEC, most of the top energy-contributing foods contributed heavily to only one macronutrient, such as nuts, ice cream, and fruit juice.

With macronutrients concentrated in a few common foods, there would be little variability in the intake of macronutrients that was not explained by energy intake, and this reduction in variability in the energy-adjusted macronutrient intake variables from the FFQ would limit the ability to determine its association with the values from the food records. On the other hand, the 142-item FFQ in this study ranks participants by their (absolute) nutrient intake reasonably well, unlike many FFQs.

Our recruitment goal was to obtain 100 subjects who would complete at least 1 day of food records plus the FFQ. This is considered a minimum number of subjects for a validation study (12), although many such studies have fewer than 100 participants (32–35). We achieved our goal by recruiting 109 participants. A larger sample size would have allowed more stable estimates of concordance by sex and race/ethnic group.

A limitation of our study is the availability of 1-day or 2-day records, which do not represent long-term intake. Subject burden and funding limitations often limit the number of days of food records that participants in validation studies can complete. However, several validation studies have used only 1–2 days of dietary data to validate an FFQ (32, 34–36). A recent review of comparisons between self-reported dietary instruments and recovery biomarkers shows that the correlations were similar using one to three dietary recalls/records (37). In addition, having 2 days of records allowed for adjustment of the correlations for day-to-day variability in intake, and the limited number of records would likely bias the concordance estimates toward the null, as infrequently consumed foods with high levels of specific nutrients, such as mango for beta-carotene or nuts for monounsaturated fat, could have been missed in the diet records.

In addition to validating a new FFQ, this study is useful more broadly for researchers and policy makers in Guam, as well as researchers developing FFQs. This is one of the few studies that documents dietary intake of adults in Guam, giving an indication of dietary food patterns. Data reported in this study can be used by health professionals and researchers on Guam to advocate for funding in nutrition education and chronic disease prevention. In addition, we demonstrate how the validation process itself can be useful to improve the FFQ in the targeted population. Using the results of the study, we will be able to improve the function of the FFQ by adding questions on the overall consumption of foods with duplicate items, such as chicken and mixed dishes, and by administration or careful review of self-administered FFQs by interviewers, especially for men and Filipinos.

In conclusion, this FFQ is sufficiently reliable and relatively valid for use in future studies that will analyze associations between diet and chronic disease. However, we have identified methods to improve the functionality of the FFQ, both at administration and for future versions. Further calibration studies are needed to improve relative validity of the FFQ's ability to better capture the long-term diet trends of the diverse population of the western Pacific, both within and beyond Guam. This FFQ will continue to be useful in helping to identify dietary intakes of this population in order to find ways to improve the health and wellness of Mariana Islanders.
